# Visit-to-visit variability in blood pressure and the risk of open-angle glaucoma in individuals without systemic hypertension: a nationwide population-based cohort study

**DOI:** 10.3389/fmed.2023.1300778

**Published:** 2024-01-10

**Authors:** Sang Yeop Lee, Ji Sung Lee, Jae Yong Kim, Hungwon Tchah, Hun Lee

**Affiliations:** ^1^Department of Ophthalmology, Yongin Severance Hospital, Yonsei University College of Medicine, Yongin, Republic of Korea; ^2^Institute of Vision Research, Department of Ophthalmology, Yonsei University College of Medicine, Seoul, Republic of Korea; ^3^Clinical Research Center, Asan Institute for Life Sciences, Asan Medical Center, University of Ulsan College of Medicine, Seoul, Republic of Korea; ^4^Department of Clinical Epidemiology and Biostatistics, Asan Medical Center, University of Ulsan College of Medicine, Seoul, Republic of Korea; ^5^Department of Ophthalmology, Asan Medical Center, University of Ulsan College of Medicine, Seoul, Republic of Korea

**Keywords:** blood pressure, blood pressure variability, KNHIS-HEALS, open-angle glaucoma, systemic hypertension

## Abstract

**Purpose:**

We aimed to evaluate the effect of visit-to-visit variability in blood pressure (BP) on the risk of open-angle glaucoma (OAG) in individuals without systemic hypertension using a population-based retrospective cohort study design.

**Methods:**

The Korean National Health Insurance Service-National Health Screening Cohort database, which collected data of 209,226 individuals between 2002 and 2015, was used to analyze the data of 140,910 eligible participants. The mean follow-up duration was 8.3 years. Visit-to-visit BP variability was assessed using standard deviation (SD), coefficient of variation (CV), and variability independent of the mean (VIM). Participants were categorized into four groups according to BP variability quartiles. We verified the effect of BP variability by comparing participants of the first to third quartiles of BP variability groups with those belonging to the fourth quartile group. A Cox proportional hazards model was used to determine the hazard ratio (HR) of BP variability in cases of newly diagnosed OAG. Moreover, we conducted subgroup analyses using baseline characteristics.

**Results:**

In the multivariable analyses, BP variability did not significantly increase the risk of OAG development. However, subgroup analyses revealed significant interactions between age and systolic BP variability in the development of OAG (CV: *p* = 0.008; SD: *p* = 0.007). For participants aged <60 years, the risk of OAG development significantly increased with high systolic BP variability (CV: HR, 1.18; 95% confidence interval [CI], 1.00–1.39; *p* = 0.049). We observed a similar trend using the SD and VIM as the parameters for systolic BP variability.

**Conclusion:**

Higher visit-to-visit systolic BP variability was associated with an increased risk of OAG development in participants younger than 60 years of age without systemic hypertension. These results suggest that BP variability can be the considerable factor when assessing the risk of OAG, especially in relatively young people without systemic hypertension.

## Introduction

Increased intraocular pressure (IOP) is a major risk factor, amongst several which are associated with the development of glaucoma (1, 2). Considering the multifactorial nature of glaucoma, its pathogenesis cannot be elucidated by the mechanical theory alone, which states that the increased IOP induces damage to the lamina cribrosa and retinal ganglion cell axons. In addition to this theory, perfusion abnormalities and vascular damage of the optic nerve head are also important mechanisms underlying the pathogenesis of glaucoma (3, 4). Arterial, venous, and intraocular pressures determine the perfusion pressure of the optic nerve head (5); thus, it is likely that blood pressure (BP) has an important role in the development and progression of glaucoma.

BP and glaucoma display varied associations owing to the complex correlation between IOP, BP, and ocular blood perfusion pressure (6). Some studies (7, 8) have demonstrated a positive correlation between BP and IOP, while others (9–11) have found that low BP correlated with optic nerve damage. One study (12), which analyzed the US National Health and Nutrition Examination Survey data, reported a nonlinear (i.e., U-shaped) relationship between the prevalence of glaucoma and systolic BP (SBP) or diastolic BP (DBP). The aforementioned relationship provides evidence for the correlation between high and low BPs and glaucoma. In addition, a high risk of open-angle glaucoma (OAG) development in patients with systemic hypertension provides evidence of the close relationship between BP and glaucoma (13).

BP does not have a constant value and changes with time. BP variability increases the risk of cardiovascular or cerebrovascular diseases such as arterial fibrillation, stroke, and dementia (14–16). Mortality in patients with diabetes is associated with BP variability (17). Considering the association between the perfusion pressure of the optic nerve head and glaucoma development, BP variability and glaucoma are likely to be associated. Lee et al. revealed that SBP variability was associated with the development of primary OAG, based on visit-to-visit BP data in a population-based cohort (18). Another population-based study using a continuous BP measurement method demonstrated a correlation between normal-tension glaucoma and BP variability (19). Thus, BP variability may also be an important factor in the development of glaucoma.

It is important to note that the aforementioned studies included participants with systemic hypertension. Systemic hypertension and the use of antihypertensive medications can be major confounding factors, thereby necessitating additional studies to determine the effect of BP variability on glaucoma development in participants without systemic hypertension. Therefore, we aimed to investigate the association between visit-to-visit BP variability and the incidence of OAG in participants without systemic hypertension by using large population-based data from the Korean National Health Insurance Service (KNHIS)-National Health Screening Cohort (HEALS).

## Methods

### Study design and population

This population-based retrospective cohort study used data from the KNHIS-HEALS database between 2002 and 2015. The health insurance system in South Korea is characterized by a nationwide, single-payer system managed by the KNHIS, which covers >98% of all South Koreans (20). It provides detailed information on age, sex, general health information, and disease diagnoses using the Korean Standard Classification of Disease (KCD) codes, similar to those of the International Classification of Disease (ICD). In addition, it provides data on prescribed medications and hospital visit history by exchanging all cost-related healthcare information between the KNHIS and medical providers using the electronic codes of the Korean Electronic Data Interchange medical procedures. All Koreans aged >40 years are eligible for the KNHIS health screening program at least once every 2 years (21). The KNHIS-HEALS database comprises publicly open data. Thus, the Institutional Review Board of the Asan Medical Center (Seoul, Korea) and the University of Ulsan College of Medicine (Seoul, Korea) approved a waiver to review the data for this study (2020–1713). This study was conducted according to the ethical principles outlined in the Declaration of Helsinki. The requirement for obtaining informed consent was waived.

We used a database comprising 209,226 Koreans who underwent health screening examinations in 2007 (i.e., the index year). We identified 177,668 participants who underwent three or more health examinations from January 1, 2002 to December 31, 2007. We excluded individuals with pre-existing OAG or angle-closure glaucoma (KCD codes H401 and H402, respectively), conditions that could cause secondary glaucoma (Supplementary Table S7), and hypertension up to the index year. Systemic hypertension was identified by the presence of ICD-10 clinical modification codes I10-I13 and I15, with a claim for the prescription of antihypertension medications. Since the first Korean hypertension diagnosis and management guideline was suggested by the Korean Society of Hypertension in 2000, the guideline underwent multiple amendments. South Korea’s very first diagnostic and treatment criteria were derived from the recommendations of the Joint National Committee on Detection, Evaluation, and Treatment of High Blood Pressure (JNC), and later adapted to account for the epidemiological characteristics of South Korean population. However, the cut-off line for diagnosing hypertension by Korean hypertension diagnosis and treatment guideline still remains to be SBP ≥ 140 mmHg or DBP ≥ 90 mmHg even in the most recent version in 2022. After excluding participants with missing data for one or more variables, a total of 140,910 participants were ultimately included in the final analysis.

### Measurements of variables and comorbidities

Health screening examination data provided the results of laboratory tests and questionnaires on health behavior, along with anthropometric details such as height, weight, and waist circumference. Moreover, the database includes BP measurements. BMI is calculated as weight in kilograms divided by the squared height in meters (kg/m^2^). Samples for the measurement of fasting serum glucose, total cholesterol, aspartate aminotransferase, alanine aminotransferase, and gamma glutamyl transferase levels were collected through venous blood sampling after an overnight fast of more than 8 h. A diagnosis of diabetes mellitus was defined as follows: (1) fasting glucose level ≥ 126 mg/dL or (2) having at least one claim per year under ICD-10 codes E10-14 and a prescription for antidiabetic medications. Dyslipidemia was defined according to the following: (1) total cholesterol ≥240 mg/dL or (2) having at least one claim per year under ICD-10 code E78 with a prescription for lipid-lowering agents. Cataract was defined, based on the ICD-10 codes related to cataract (i.e., H25.0, H25.1, H25.2, H25.8, H25.9, H26.02, H26.21, H26.28, H26.3, H26.8, H26.9, H28.0, H28.1, and H28.2). We used the Charlson Comorbidity Index as a covariate to match the overall general health statuses between the groups. This score refers to a weighted index calculated by the presence of 17 systemic diseases. Thus, the higher the score, the greater the burden of systemic diseases (22). Current smoking status, alcohol consumption, regular exercise, and income level were defined by the questionnaire results. The response to cigarette smoking was “never,” “ex-smoker,” or “current smoker.” In the present study, a current smoker was defined as a response of “current smoker” among the aforementioned three items. Alcohol consumption was defined as drinking 3–4 times a week or more, whereas regular exercise was defined as exercising five times or more a week. Low household income level was defined as an income level of <10%.

### BP measurements and visit-to-visit BP variability

During the KNHIS health screening examination, a trained clinician measured brachial BP according to the protocol using a standardized sphygmomanometer. This measurement was conducted twice in participants after a five-minute rest period and their average BP was recorded. SBP and DBP were measured separately. Moreover, BP variability was assessed using the SD, CV, and VIM of BP until the index year. The CV was defined as the SD divided by the mean value, whereas the VIM was calculated as follows: 100 × SD/mean^β^ (23), where *β* refers to the regression coefficient based on the natural logarithm of the SD over the natural logarithm of the mean. The number of BP measurements ranged from three to six per individual, as follows: three measurements (*n* = 68,698); four measurements (*n* = 15,436); five measurements (*n* = 21,207), and six measurements (*n* = 35,749).

### Study outcomes and follow-up

Newly diagnosed OAG was the primary outcome. OAG development was defined when the following three criteria were met: (1) a diagnosis of OAG, based on the KCD code (H401), (2) undergoing a visual field test more than once, and (3) a prescription for antiglaucoma medications (13). We followed the cohort from the index date until the date of being newly diagnosed with OAG or until the end of the study (December 21, 2015), whichever occurred first. The mean and median follow-up duration was 8.2 years and 8.4 years, respectively. The interquartile range of the follow-up duration was 8.2–8.6 years.

### Statistical analysis

Participants were classified into four groups based on the BP variability quartile. Differences in the distribution of baseline characteristics among the BP quartile groups were identified by using analysis of variance or the Chi-square test, as appropriate. We used the Cox proportional hazards model to estimate the hazard ratio (HR) and the 95% confidence interval (CI) of newly diagnosed OAG. Multivariable analyses were conducted using two models, based on the type of variables used for the adjustment. Model 1 was adjusted for age and sex; model 2 was adjusted for all variables used to demonstrate the baseline characteristics. We conducted subgroup analyses within the baseline characteristics for a better understanding of the effect of BP variability on newly diagnosed OAG. Therefore, the interaction effect between BP variability and each subgroup was evaluated using Cox regression analysis. A two-sided *p*-value of <0.05 was considered statistically significant. All statistical analyses were conducted using SAS 9.4 (SAS Institute, Cary, NC, USA).

## Results

### Baseline characteristics

Tables 1, 2 summarize the characteristics of the study participants, based on their variability independent of the mean (VIM) quartiles for SBP and DBP. Significant differences were observed in all variables among the VIM SBP groups, except for fasting glucose level (*p* = 0.679). In contrast, among the VIM DBP groups, significant differences were observed in all variables, except for fasting glucose level (*p* = 0.304) and alcohol consumption (*p* = 0.199). We observed similar results with defining BP variability by the standard deviation (SD) and coefficient of variation (CV) of BP (Supplementary Tables S1–S4). Upon defining SBP and DBP variabilities as SD, we observed a significant difference among the quartile groups for all variables. Upon defining SBP variability by the CV, all variables, except for the total cholesterol level, demonstrated significant differences among the quartile groups. In contrast, significant differences were observed in all variables, except for fasting glucose level and alcohol consumption, on defining diastolic BP variability by CV, which was similar to the VIM results.

**Table 1:** Baseline characteristics of the study participants, based on the VIM quartiles for visit-to-visit variability of systolic blood pressure.

	Q1	Q2	Q3	Q4	*P*-value ^a^
*N*	35,246	35,209	35,228	35,227	
Age (y)	55.2 ± 8.5	54.3 ± 7.9	54.8 ± 8.2	56.8 ± 9.1	<0.0001
Sex (male)	21,064 (59.8)	22,510 (63.9)	21,775 (61.8)	18,550 (52.7)	<0.0001
BMI (kg/m^2^)	24.0 ± 2.8	23.9 ± 2.8	23.8 ± 2.8	23.8 ± 2.9	<0.0001
Mean SBP	126.2 ± 11.9	125.5 ± 12.7	124.8 ± 12.5	124.6 ± 13.9	<0.0001
Mean DBP	79.1 ± 7.7	78.9 ± 8.1	78.4 ± 7.9	77.9 ± 8.5	<0.0001
SBP variability					
CV (%)	3.7 ± 1.4	6.6 ± 0.9	9.0 ± 1.0	13.7 ± 3.2	<0.0001
SD	4.7 ± 1.9	8.3 ± 1.7	11.3 ± 2.2	17.2 ± 5.2	<0.0001
VIM	4.7 ± 1.8	8.3 ± 0.9	11.5 ± 1.0	17.5 ± 3.8	<0.0001
DBP variability					
CV (%)	7.1 ± 3.8	8.2 ± 3.8	9.5 ± 4.0	12.4 ± 5.2	<0.0001
SD	5.6 ± 3.1	6.4 ± 3.0	7.5 ± 3.3	9.7 ± 4.4	<0.0001
VIM	5.6 ± 3.1	6.5 ± 3.0	7.6 ± 3.2	9.9 ± 4.1	<0.0001
FPG	97.5 ± 23.3	97.4 ± 23.1	97.6 ± 24.0	97.6 ± 23.8	0.679
Total cholesterol	199.3 ± 36.3	198.6 ± 36.0	198.6 ± 36.1	198.7 ± 37.0	0.028
AST	26.0 ± 14.2	26.1 ± 15.3	26.2 ± 16.6	26.4 ± 16.4	0.007
ALT	25.2 ± 18.5	25.5 ± 19.0	25.2 ± 20.9	24.7 ± 19.9	<0.0001
GGT	38.2 ± 47.9	39.0 ± 49.0	39.2 ± 52.2	37.7 ± 52.5	0.0001
DM	4,966 (14.1)	5,324 (15.1)	5,438 (15.4)	5,545 (15.7)	<0.0001
Hyperlipidemia	11,461 (32.5)	11,351 (32.2)	11,504 (32.7)	11,953 (33.9)	<0.0001
Cataract	2,723 (7.7)	2,282 (6.5)	2,384 (6.8)	3,216 (9.1)	<0.0001
CCI					<0.0001
0	13,744 (39.0)	14,326 (40.7)	13,936 (39.6)	12,241 (34.7)	
1	9,922 (28.2)	9,930 (28.2)	9,846 (27.9)	9,891 (28.1)	
2	5,649 (16.0)	5,510 (15.6)	5,837 (16.6)	6,139 (17.4)	
≥3	5,931 (16.8)	5,443 (15.5)	5,609 (15.9)	6,956 (19.7)	
Current smoker	7,055 (20.0)	8,036 (22.8)	7,835 (22.2)	6,770 (19.2)	<0.0001
Alcohol consumption	3,455 (9.8)	3,504 (10.0)	3,555 (10.1)	3,280 (9.3)	0.003
Regular exercise	3,675 (10.4)	3,347 (9.5)	3,369 (9.6)	3,531 (10.0)	<0.0001
Income (<10%)	2,493 (7.1)	2,555 (7.3)	2,813 (8.0)	3,233 (9.2)	<0.0001

Data are expressed as the mean ± the SD or as *n* (%).

a The *P*-value is derived from the analysis of variance and the Chi-square test.

VIM, variability independent of the mean; N, number; Q1–4, quartile 1–4; BMI, body mass index; SBP, systolic blood pressure; DBP, diastolic blood pressure; CV, coefficient of variation; SD, standard deviation; FPG, fasting plasma glucose; AST, aspartate aminotransferase; ALT, alanine aminotransferase; GGT, gamma glutamyl transferase; DM, diabetes mellitus; and CCI, Charlson Comorbidity Index.

**Table 2:** Baseline characteristics of the study participants, based on the VIM quartiles for visit-to-visit variability of diastolic blood pressure.

	Q1	Q2	Q3	Q4	*P*-value ^a^
*N*	35,228	35,224	35,232	35,226	
Age (y)	55.2 ± 8.3	54.9 ± 8.4	54.4 ± 7.9	56.6 ± 9.1	<0.0001
Sex (male)	21,003 (59.6)	21,428 (60.8)	22,131 (62.8)	19,337 (54.9)	<0.0001
BMI (kg/m^2^)	24.0 ± 2.8	23.8 ± 2.8	23.8 ± 2.8	23.8 ± 2.9	<0.0001
Mean SBP	126.3 ± 12.4	124.3 ± 11.7	124.9 ± 13.0	125.7 ± 13.8	<0.0001
Mean DBP	79.5 ± 7.9	78.1 ± 7.2	78.4 ± 8.4	78.3 ± 8.7	<0.0001
SBP variability					
CV (%)	6.3 ± 3.2	7.2 ± 3.3	8.4 ± 3.5	11.0 ± 4.7	<0.0001
SD	8.0 ± 4.2	9.0 ± 4.3	10.5 ± 4.7	14.0 ± 6.6	<0.0001
VIM	8.0 ± 4.0	9.3 ± 4.2	10.7 ± 4.4	14.0 ± 5.9	<0.0001
DBP variability					
CV (%)	4.0 ± 2.0	7.4 ± 0.6	10.2 ± 1.0	15.6 ± 3.4	<0.0001
SD	3.2 ± 1.6	5.8 ± 0.7	8.0 ± 1.2	12.2 ± 3.2	<0.0001
VIM	3.2 ± 1.6	5.9 ± 0.5	8.1 ± 0.8	12.3 ± 2.7	<0.0001
FPG	97.7 ± 22.6	97.5 ± 24.2	97.4 ± 23.8	97.6 ± 23.6	0.304
Total cholesterol	199.7 ± 36.2	198.6 ± 36.1	198.2 ± 36.1	198.7 ± 36.9	<0.0001
AST	26.2 ± 15.7	26.0 ± 15.2	26.2 ± 15.6	26.3 ± 16.1	0.043
ALT	25.5 ± 20.4	25.1 ± 18.5	25.4 ± 20.0	24.7 ± 19.6	<0.0001
GGT	38.6 ± 49.2	37.8 ± 48.5	39.2 ± 49.9	38.5 ± 53.9	0.004
DM	5,067 (14.4)	5,102 (14.5)	5,479 (15.6)	5,625 (16.0)	<0.0001
Hyperlipidemia	11,471 (32.6)	11,225 (31.9)	11,504 (32.7)	12,069 (34.3)	<0.0001
Cataract	2,674 (7.6)	2,479 (7.0)	2,307 (6.5)	3,145 (8.9)	<0.0001
CCI					<0.0001
0	13,752 (39.0)	13,887 (39.4)	14,118 (40.1)	12,490 (35.5)	
1	9,837 (27.9)	9,921 (28.2)	10,014 (28.4)	9,817 (27.9)	
2	5,784 (16.4)	5,601 (15.9)	5,637 (16.0)	6,113 (17.4)	
≥3	5,855 (16.6)	5,815 (16.5)	5,463 (15.5)	6,806 (19.3)	
Current smoker	7,176 (20.4)	7,591 (21.6)	8,014 (22.7)	6,915 (19.6)	<0.0001
Alcohol consumption	3,526 (10.0)	3,488 (9.9)	3,388 (9.6)	3,392 (9.6)	0.199
Regular exercise	3,608 (10.2)	3,479 (9.9)	3,337 (9.5)	3,498 (9.9)	0.008
Income (<10%)	2,586 (7.3)	2,555 (7.3)	2,806 (8.0)	3,147 (8.9)	<0.0001

Data are expressed as the mean ± the SD or as *n* (%).

a The *P*-value is derived from the analysis of variance and the Chi-square test.

VIM, variability independent of the mean; N, number; Q1–4, quartile 1–4; BMI, body mass index; SBP, systolic blood pressure; DBP, diastolic blood pressure; CV, coefficient of variation; SD, standard deviation; FPG, fasting plasma glucose; AST, aspartate aminotransferase; ALT, alanine aminotransferase; GGT, gamma glutamyl transferase; DM, diabetes mellitus; and CCI, Charlson Comorbidity Inde.

### Effect of BP variability on the development of OAG

Table 3 summarizes the HRs and 95% CIs of newly diagnosed OAG according to BP variability (per 10-unit increase). Only a significant increase in the risk of OAG development correlated with SBP variability, defined by SD (HR, 1.13; 95% CI, 1.03–1.24). However, multivariable analyses revealed no significant findings in any BP variability parameter. As an additional method to verify the effect of BP variability, we compared the risk of OAG development in the highest quartile group (Q4) with that in the lower three quartiles (Q1–Q3) as the reference group (Table 4). Without an adjustment for the variables, we observed a significantly increased risk of OAG development in the Q4 group, compared with the Q1–Q3 group in all three parameters for BP variability. However, BP variability did not significantly increase the risk of OAG development in model 1 and model 2 analyses that adjusted for the variables.

**Table 3:** Hazard ratios and 95% confidence intervals of newly diagnosed open angle glaucoma, based on visit-to-visit variability of blood pressure (per 10-unit increase).

BP variability (per 10-unit increase)	Unadjusted		Model 1		Model 2	
	HR (95% CI)	*P*-value	HR (95% CI)	*P*-value	HR (95% CI)	*P*-value
SBP						
CV	1.12 (0.99–1.27)	0.076	0.96 (0.85–1.09)	0.525	0.96 (0.85–1.08)	0.477
SD	1.13 (1.03–1.24)	0.008	0.97 (0.89–1.06)	0.536	0.96 (0.87–1.05)	0.387
VIM	1.05 (0.95–1.16)	0.318	0.97 (0.88–1.07)	0.496	0.97 (0.88–1.07)	0.518
DBP						
CV	1.10 (0.99–1.23)	0.086	1.03 (0.92–1.14)	0.642	1.02 (0.92–1.13)	0.718
SD	1.14 (1.00–1.30)	0.052	1.02 (0.90–1.16)	0.737	1.02 (0.89–1.16)	0.802
VIM	1.13 (0.98–1.29)	0.091	1.03 (0.90–1.18)	0.636	1.03 (0.90–1.17)	0.713

Model 1 is adjusted for age and sex. Model 2 is adjusted for the variables in Model 1 + the other variables in Table 1.

OAG, open-angle glaucoma; BP, blood pressure; HR, hazard ratio; SBP, systolic blood pressure; DBP, diastolic blood pressure; VIM, variability independent of the mean; CV, coefficient of variation; and SD, standard deviation; VIM, variability independent of the mean.

**Table 4:** Hazard ratios and 95% confidence intervals of newly diagnosed OAG with comparison between quartile 1–3 and quartile 4 of visit-to-visit variability of blood pressure.

				Unadjusted		Model 1		Model 2	
	Events (n)	F/U duration (person-years)	Incidence (95% CI)	HR (95% CI)	*P*-value	HR (95% CI)	*P*-value	HR (95% CI)	*P*-value
SBP variability									
CV									
Q1–Q3	1,027	872,203	1.18 (1.11–1.25)	1 (ref)		1 (ref)		1 (ref)	
Q4	394	288,432	1.37 (1.24–1.51)	1.16 (1.03–1.30)	0.012	1.01 (0.90–1.14)	0.866	1.00 (0.89–1.13)	0.958
SD									
Q1–Q3	1,016	872,710	1.16 (1.09–1.24)	1 (ref)		1 (ref)		1 (ref)	
Q4	405	287,926	1.41 (1.28–1.55)	1.21 (1.08–1.36)	0.001	1.01 (0.90–1.13)	0.900	0.99 (0.88–1.12)	0.904
VIM									
Q1–Q3	1,022	871,817	1.17 (1.10–1.25)	1 (ref)		1 (ref)		1 (ref)	
Q4	399	288,818	1.38 (1.25–1.52)	1.18 (1.05–1.32)	0.006	1.06 (0.95–1.20)	0.291	1.06 (0.95–1.19)	0.306
DBP variability									
CV									
Q1–Q3	1,025	871,536	1.18 (1.11–1.25)	1 (ref)		1 (ref)		1 (ref)	
Q4	396	289,099	1.37 (1.24–1.51)	1.16 (1.04–1.31)	0.010	1.06 (0.95–1.19)	0.309	1.06 (0.94–1.19)	0.352
SD									
Q1–Q3	1,018	872,155	1.17 (1.10–1.24)	1 (ref)		1 (ref)		1 (ref)	
Q4	403	288,480	1.40 (1.27–1.54)	1.20 (1.07–1.34)	0.002	1.06 (0.94–1.19)	0.326	1.06 (0.94–1.19)	0.373
VIM									
Q1–Q3	1,028	871,770	1.18 (1.11–1.25)	1 (ref)		1 (ref)		1 (ref)	
Q4	393	288,865	1.36 (1.23–1.50)	1.15 (1.03–1.30)	0.016	1.05 (0.94–1.18)	0.390	1.05 (0.93–1.18)	0.442

Model 1 is adjusted for age and sex. Model 2 is adjusted for the variables in Model 1 + the other variables in Table 1.

OAG, open-angle glaucoma; BP, blood pressure; F/U, follow-up; HR, hazard ratio; CI, confidence interval; SBP, systolic blood pressure; CV, coefficient of variation; Q1–4, quartile 1–4; SD, standard deviation; VIM, variability independent of the mean; DBP, diastolic blood pressure.

### Subgroup analyses

We conducted stratified analyses by age, sex, body mass index (BMI), diabetes mellitus, hyperlipidemia, cataract, smoking status, alcohol consumption status, regular exercise status, and income status. Table 5 summarizes the results of the stratified analyses for the effect of SBP variability on OAG development according to the CV. Based on the subgroup analysis of factors that could affect this development, we identified a significant interaction for age × SBP variability (CV, SD) (*p* = 0.009 for CV and *p* = 0.007 for SD) (Table 5; Supplementary Table S5). The risk of OAG development significantly increased with high SBP variability (CV) for participants aged <60 years (HR, 1.18; 95% CI, 1.00–1.39; *p* = 0.0485). We observed a similar trend when using SD and VIM as the parameters for SBP variability (SD: HR, 1.18; 95% CI, 0.99–1.39; *p* = 0.061; VIM: HR, 1.18; 95% CI, 1.00–1.39; *p* = 0.048) (Supplementary Tables S5, S6). However, no condition significantly affected the relationship between DBP variability and the risk of OAG development.

**Table 5:** Subgroup analyses for the effect of visit-to-visit variability of systolic blood pressure (CV) on the development of open angle glaucoma.

	Q1–Q3	Q4	HR (95% CI)	*P*-value	*P* for interaction
Age					0.008
<60 y	563/79689	191/22333	1.18 (1.00–1.39)	0.049	
≥60 y	464/25990	203/12898	0.86 (0.73–1.01)	0.073	
Sex					0.546
Female	402/40923	172/16088	0.96 (0.80–1.15)	0.676	
Male	625/64756	222/19143	1.03 (0.89–1.21)	0.664	
BMI (kg/m^2^)					0.395
<25	689/71711	252/23388	0.97 (0.84–1.12)	0.649	
≥25	338/33968	142/11843	1.07 (0.88–1.31)	0.475	
DM					0.639
No	856/90430	313/29207	0.99 (0.87–1.13)	0.878	
Yes	171/15249	81/6024	1.06 (0.81–1.38)	0.656	
Hyperlipidemia					0.921
No	657/71784	240/22857	1.00 (0.86–1.16)	0.984	
Yes	370/33895	154/12374	1.01 (0.84–1.22)	0.912	
Cataract					0.411
No	869/98412	324/31893	1.03 (0.90–1.17)	0.695	
Yes	158/7267	70/3338	0.90 (0.68–1.19)	0.470	
Current smoker					0.230
No	836/82839	338/28375	1.04 (0.91–1.18)	0.595	
Yes	191/22840	56/6856	0.85 (0.63–1.14)	0.283	
Alcohol consumption					0.923
No	920/95426	351/31690	1.00 (0.88–1.13)	0.985	
Yes	107/10253	43/3541	1.02 (0.72–1.45)	0.914	
Regular exercise					0.564
No	886/95360	344/31628	1.02 (0.90–1.15)	0.795	
Yes	141/10319	50/3603	0.92 (0.66–1.27)	0.606	
Income (<10%)					0.956
No	940/97923	353/31893	1.00 (0.89–1.14)	0.946	
Yes	87/7756	41/3338	0.99 (0.68–1.44)	0.971	

Hazard ratios were calculated based on multivariate Cox regression after being adjusted for variables belonging to model 2.

Data are expressed as the number of events/number of patients. Model 1 is adjusted for age and sex. Model 2 is adjusted for the variables in Model 1 + the other variables in Table 1.

BP, blood pressure; OAG, open-angle glaucoma; CV, coefficient of variation; Q1–4, quartile 1–4; HR, hazard ratio; CI, confidence interval; BMI, body mass index; DM, diabetes mellitus.

## Discussion

In the present study, we investigated the association between visit-to-visit BP variability and OAG development in participants without systemic hypertension by using a large longitudinal population-based cohort. BP variability significantly increased the risk of OAG development, according to the unadjusted analyses; however, this finding was not confirmed after adjusting for the variables. In the subgroup analyses, the visit-to-visit SBP variability, which was defined using three different indicators, was associated with an increased risk of OAG development in participants aged <60 years without systemic hypertension. Previously, researchers have reported the association between BP variability and the development of primary OAG using the National Sample Cohort of the KNHIS (18). Moreover, there are reports which verify the association between BP variability and glaucoma (19, 24). However, these studies included participants with systemic hypertension. Thus, confounding factors related to BP variability, such as the use of antihypertensive medications, would likely affect the results. Unlike previous studies, only data of participants without systemic hypertension were analyzed in this study. The difference in the inclusion of participants with systemic hypertension in previous studies may explain why our subgroup analysis demonstrated that SBP variability induced a significantly increased risk of OAG development.

Under physiological condition, BP naturally fluctuates, which is essential for maintaining appropriate multiple-organ perfusion. Therefore, failure to control BP fluctuations results in a high possibility of impaired organ function. Longitudinal and cross-sectional studies have reported that an increase in the visit-to-visit BP variability is associated with a greater risk of organ damage, cardiovascular events, and mortality (16, 25, 26). In addition, researchers have verified its association with the occurrence of neurodegenerative diseases such as dementia and Alzheimer’s disease (15, 27, 28). Nonetheless, investigators have not clearly elucidated the mechanism underlying the effect of BP variability on various pathologic conditions, including OAG. Considering the involvement of vascular dysregulation in the pathophysiology of glaucoma (29, 30), the association between BP variability and cardiovascular homeostasis is supposedly a key factor that explains the correlation between glaucoma development and BP variability (16). Arterial stiffness is another important factor in the relationship between BP variability and glaucoma. Arterial stiffness has an important role in BP variability (31). A recent study using optical coherence tomography angiography verified that a high pulse-wave velocity was associated with decreased macular vessel density in patients with normal-tension glaucoma (32). Pulse-wave velocity is a representative parameter of arterial stiffness. Therefore, the aforementioned result indicates a possible correlation between arterial stiffness and the pathogenesis of normal-tension glaucoma. Autonomic dysfunction may also play a role in the relationship between BP variability and glaucoma; it induces BP variability and reduces ocular perfusion pressure (33–36). Although there is insufficient data from previous studies regarding the exact mechanism, it is not difficult to conclude that a relationship exists between BP variability and glaucoma is related to vascular dysregulation. Further studies are warranted to directly verify the relationship between BP variability and ocular blood supply and the mechanism underlying the effect of BP variability on glaucoma development, particularly with the use of devices such as coherence tomography angiography. Nevertheless, our study is of sufficient value in that it supports the vascular theory underlying the pathogenesis of glaucoma and identified clinical factors associated with OAG development.

In the present study, the age of the Q4 group was higher than that of the other groups. In addition, in the subgroup analyses, the Q4 group included a relatively higher number of participants aged ≥60 years than participants aged <60 years. These results are similar to those of previous studies demonstrating that BP variability increases with age (37, 38). Many of the possible underlying mechanisms associated with aging, such as hemodynamic instability, advanced arterial remodeling, atherosclerosis, arterial stiffness, baroreflex impairment, endothelial dysfunction, and subclinical inflammation, are interconnected (39). Although further investigations are necessary to establish whether elevated blood pressure variability constitutes a hallmark of aging, it has recently garnered attention as a potential candidate marker for aging, owing to its associations with the mechanisms mentioned above. Age-related impairment to the baroreflex or an increase in arterial stiffness is the primary mechanism underlying the relationship between BP variability and age (38). Therefore, an increase in BP variability at a relatively young age indicates a more pathological condition, unlike age-related changes, which may be a factor that increases susceptibility to organ damage. SBP variability exerts a greater effect on stroke risk at a young age (23). Glaucoma requires an early diagnosis and lifelong treatment to reduce the likelihood of progression of visual function impairment. The development of glaucoma at a young age increases the likelihood of encountering various problems caused by the progression of visual function impairment. Therefore, the finding of the subgroup analysis that large SBP variability significantly increased the risk of OAG development in relatively young participants without systemic hypertension has clinical significance. Investigators should perform additional research, such as determining clinical factors affecting the impact of SBP variability on the risk of OAG development with age to accurately verify the relationship between them. However, our findings likely have clinical significance in that they identified a notable factor to be considered (i.e., BP variability), while classifying and monitoring a high-risk group for OAG.

The following limitations of the current study should be considered when interpreting these results. First, determining the presence of a disease based on diagnostic codes is a representative limitation of studies using claim data. To minimize possible diagnostic inaccuracies, we used additional parameters, such as medication use and clinical examination. Second, we only included participants who had visited a hospital. Thus, we cannot exclude the possibility of underestimating the presence of OAG and other diseases. Third, there was no control of the BP measurement conditions, which we presumed was not performed accurately. This is a common limitation of previous studies that verified a relationship between visit-to-visit BP variability and pathologic conditions using KNHIS data. However, BP measurements obtained during health screening examinations, based on the protocol of medical institutions certified by the KNHIS, alleviated the risk of measurement bias. Fourth, the BP variability was based on a range of three to six BP measurements per subjects. Furthermore, the temporal intervals between BP measurements exhibited non-uniformity. In investigating visit-to-visit BP variability, it is important to define the BP measurement interval and number of BP measurements. Given the characteristics of the data collected for this research, it is unfeasible to obtain BP measurements at consistent intervals or at the same frequency. Nonetheless, it is noteworthy that the methodology employed for characterizing BP variability in our study has been consistently utilized in several prior studies (14, 18, 40, 41) that defined BP variability using KNHIS data. Fifth, our findings were obtained from a database with data for an overwhelmingly large population of Koreans. This factor warrants cautiously applying the present findings to other ethnic groups. Finally, despite using longitudinal follow-up results with a large sample size, our study was based on a retrospective design. Therefore, our findings should be confirmed by additional prospective longitudinal studies that include other ethnic groups.

In conclusion, high visit-to-visit SBP variability significantly increased the risk of OAG development in participants aged <60 years; however, our results did not demonstrate an effect of high BP variability on an increased risk of OAG development for all age groups. Although it needs to be verified through further research, our results indicate that BP variability may be a factor to consider when assessing the risk of OAG development in relatively young people without systemic hypertension. In addition, the importance of BP variability in the development of glaucoma is likely to be considered as remarkable evidence that supports the vascular theory, which explains the pathophysiology of glaucoma.

## Data availability statement

The raw data supporting the conclusions of this article will be made available by the authors, without undue reservation.

## Ethics statement

The Institutional Review Board of the Asan Medical Center (Seoul, Korea) and the University of Ulsan College of Medicine (Seoul, Korea) approved a waiver to review the data for this study (2020-1713). This study was conducted according to the ethical principles outlined in the Declaration of Helsinki. The requirement for obtaining informed consent was waived. The studies were conducted in accordance with the local legislation and institutional requirements. The ethics committee/institutional review board waived the requirement of written informed consent for participation from the participants or the participants‘ legal guardians/next of kin because the KNHIS-HEALS database comprises publicly opened data.

## Author contributions

SL: Conceptualization, Data curation, Investigation, Methodology, Writing – original draft, Writing – review & editing. JL: Conceptualization, Data curation, Investigation, Methodology, Writing – review & editing, Formal analysis, Validation. JK: Conceptualization, Formal analysis, Investigation, Methodology, Writing – review & editing. HT: Conceptualization, Investigation, Methodology, Resources, Supervision, Writing – review & editing. HL: Conceptualization, Data curation, Formal analysis, Funding acquisition, Investigation, Methodology, Supervision, Validation, Writing – original draft, Writing – review & editing, Resources.

## References

[1] Jonas JB Aung T Bourne RR Bron AM Ritch R Panda-Jonas S. Glaucoma. Lancet. (2017) 390:2183–93. doi: 10.1016/s0140-6736(17)31469-1 28577860

[2] Weinreb RN Aung T Medeiros FA. The pathophysiology and treatment of glaucoma: a review. JAMA. (2014) 311:1901–11. doi: 10.1001/jama.2014.3192, PMID: 24825645 PMC4523637

[3] Flammer J Orgül S Costa VP Orzalesi N Krieglstein GK Serra LM. The impact of ocular blood flow in glaucoma. Prog Retin Eye Res. (2002) 21:359–93. doi: 10.1016/s1350-9462(02)00008-3 12150988

[4] Yanagi M Kawasaki R Wang JJ Wong TY Crowston J Kiuchi Y. Vascular risk factors in glaucoma: a review. Clin Exp Ophthalmol. (2011) 39:252–8. doi: 10.1111/j.1442-9071.2010.02455.x 20973906

[5] Costa VP Harris A Anderson D Stodtmeister R Cremasco F Kergoat H. Ocular perfusion pressure in glaucoma. Acta Ophthalmol. (2014) 92:e252–66. doi: 10.1111/aos.12298 24238296

[6] Costa VP Arcieri ES Harris A. Blood pressure and glaucoma. Br J Ophthalmol. (2009) 93:1276–82. doi: 10.1136/bjo.2008.149047 19336425

[7] Bonomi L Marchini G Marraffa M Bernardi P Morbio R Varotto A. Vascular risk factors for primary open angle glaucoma: the Egna-Neumarkt Study. Ophthalmology. (2000) 107:1287–93. doi: 10.1016/s0161-6420(00)00138-x 10889099

[8] Klein BE Klein R Knudtson MD. Intraocular pressure and systemic blood pressure: longitudinal perspective: the Beaver Dam Eye Study. Br J Ophthalmol. (2005) 89:284–7. doi: 10.1136/bjo.2004.048710, PMID: 15722304 PMC1772559

[9] Cherecheanu AP Garhofer G Schmidl D Werkmeister R Schmetterer L. Ocular perfusion pressure and ocular blood flow in glaucoma. Curr Opin Pharmacol. (2013) 13:36–42. doi: 10.1016/j.coph.2012.09.003, PMID: 23009741 PMC3553552

[10] Memarzadeh F Ying-Lai M Chung J Azen SP Varma R. Blood pressure, perfusion pressure, and open-angle glaucoma: the Los Angeles Latino eye study. Invest Ophthalmol Vis Sci. (2010) 51:2872–7. doi: 10.1167/iovs.08-2956, PMID: 20089880 PMC2891455

[11] Quigley HA West SK Rodriguez J Munoz B Klein R Snyder R. The prevalence of glaucoma in a population-based study of Hispanic subjects: Proyecto VER. Arch Ophthalmol. (2001) 119:1819–26. doi: 10.1001/archopht.119.12.1819 11735794

[12] Kim H Choi B. Nonlinear relationship between blood pressure and Glaucoma in US adults. Am J Hypertens. (2019) 32:308–16. doi: 10.1093/ajh/hpy186 30535341

[13] Rim TH Lee SY Kim SH Kim SS Kim CY. Increased incidence of open-angle glaucoma among hypertensive patients: an 11-year nationwide retrospective cohort study. J Hypertens. (2017) 35:729–36. doi: 10.1097/hjh.0000000000001225 28253217

[14] Lee SR Choi YJ Choi EK Han KD Lee E Cha MJ. Blood pressure variability and incidence of new-onset atrial fibrillation: a nationwide population-based study. Hypertension. (2020) 75:309–15. doi: 10.1161/hypertensionaha.119.13708, PMID: 31838903

[15] Ma Y Tully PJ Hofman A Tzourio C. Blood pressure variability and dementia: a state-of-the-art review. Am J Hypertens. (2020) 33:1059–66. doi: 10.1093/ajh/hpaa119, PMID: 32710605

[16] Parati G Torlasco C Pengo M Bilo G Ochoa JE. Blood pressure variability: its relevance for cardiovascular homeostasis and cardiovascular diseases. Hypertens Res. (2020) 43:609–20. doi: 10.1038/s41440-020-0421-5, PMID: 32203448

[17] Hsieh YT Tu ST Cho TJ Chang SJ Chen JF Hsieh MC. Visit-to-visit variability in blood pressure strongly predicts all-cause mortality in patients with type 2 diabetes: a 5·5-year prospective analysis. Eur J Clin Investig. (2012) 42:245–53. doi: 10.1111/j.1365-2362.2011.02574.x, PMID: 21815887

[18] Lee NY Jung Y Han K Park CK. Fluctuation in systolic blood pressure is a major systemic risk factor for development of primary open-angle glaucoma. Sci Rep. (2017) 7:43734. doi: 10.1038/srep43734, PMID: 28262703 PMC5338023

[19] Melgarejo JD Maestre GE Mena LJ Lee JH Petitto M Chávez CA. Normal-tension glaucomatous optic neuropathy is related to blood pressure variability in the Maracaibo aging study. Hypertens Res. (2021) 44:1105–12. doi: 10.1038/s41440-021-00687-1, PMID: 34253881 PMC8429242

[20] Kwon S. Thirty years of national health insurance in South Korea: lessons for achieving universal health care coverage. Health Policy Plan. (2009) 24:63–71. doi: 10.1093/heapol/czn037, PMID: 19004861

[21] Seong SC Kim YY Park SK Khang YH Kim HC Park JH. Cohort profile: the National Health Insurance Service-National Health Screening Cohort (NHIS-HEALS) in Korea. BMJ Open. (2017) 7:e016640. doi: 10.1136/bmjopen-2017-016640, PMID: 28947447 PMC5623538

[22] Charlson ME Pompei P Ales KL MacKenzie CR. A new method of classifying prognostic comorbidity in longitudinal studies: development and validation. J Chronic Dis. (1987) 40:373–83. doi: 10.1016/0021-9681(87)90171-8, PMID: 3558716

[23] Rothwell PM Howard SC Dolan E O'Brien E Dobson JE Dahlöf B. Prognostic significance of visit-to-visit variability, maximum systolic blood pressure, and episodic hypertension. Lancet. (2010) 375:895–905. doi: 10.1016/s0140-6736(10)60308-x, PMID: 20226988

[24] Lindemann F Kuerten D Koch E Fuest M Fischer C Voss A. Blood pressure and heart rate variability in primary open-angle glaucoma and normal tension glaucoma. Curr Eye Res. (2018) 43:1507–13. doi: 10.1080/02713683.2018.1506036, PMID: 30110187

[25] Mancia G Facchetti R Parati G Zanchetti A. Visit-to-visit blood pressure variability, carotid atherosclerosis, and cardiovascular events in the European Lacidipine Study on Atherosclerosis. Circulation. (2012) 126:569–78. doi: 10.1161/circulationaha.112.107565 22761453

[26] Chang TI Reboussin DM Chertow GM Cheung AK Cushman WC Kostis WJ. Visit-to-visit office blood pressure variability and cardiovascular outcomes in SPRINT (systolic blood pressure intervention trial). Hypertension. (2017) 70:751–8. doi: 10.1161/hypertensionaha.117.09788, PMID: 28760939 PMC6209591

[27] Lattanzi S Luzzi S Provinciali L Silvestrini M. Blood pressure variability in Alzheimer's disease and frontotemporal dementia: the effect on the rate of cognitive decline. J Alzheimers Dis. (2015) 45:387–94. doi: 10.3233/jad-142532, PMID: 25790932

[28] de Heus RAA Olde Rikkert MGM Tully PJ Lawlor BA Claassen J. Blood pressure variability and progression of clinical Alzheimer disease. Hypertension. (2019) 74:1172–80. doi: 10.1161/hypertensionaha.119.13664, PMID: 31542965

[29] Hayreh SS. Blood flow in the optic nerve head and factors that may influence it. Prog Retin Eye Res. (2001) 20:595–624. doi: 10.1016/s1350-9462(01)00005-2 11470452

[30] Harris A Werne A Cantor LB. Vascular abnormalities in glaucoma: from population-based studies to the clinic? Am J Ophthalmol. (2008) 145:595–7. doi: 10.1016/j.ajo.2007.12.019 18358850

[31] Shimbo D Shea S McClelland RL Viera AJ Mann D Newman J. Associations of aortic distensibility and arterial elasticity with long-term visit-to-visit blood pressure variability: the Multi-Ethnic Study of Atherosclerosis (MESA). Am J Hypertens. (2013) 26:896–902. doi: 10.1093/ajh/hpt040, PMID: 23537891 PMC3693480

[32] Lee T Bae HW Seong GJ Kim CY Lee SY. High pulse wave velocity is associated with decreased macular vessel density in normal-tension glaucoma. Invest Ophthalmol Vis Sci. (2021) 62:12. doi: 10.1167/iovs.62.10.12 PMC8374976 34398200

[33] Park HY Jung KI Na KS Park SH Park CK. Visual field characteristics in normal-tension glaucoma patients with autonomic dysfunction and abnormal peripheral microcirculation. Am J Ophthalmol. (2012) 154:466–75.e1. doi: 10.1016/j.ajo.2012.03.028, PMID: 22704139

[34] Kurysheva NI Ryabova TY Shlapak VN. Heart rate variability: the comparison between high tension and normal tension glaucoma. EPMA J. (2018) 9:35–45. doi: 10.1007/s13167-017-0124-4 29515686 PMC5833892

[35] Spallone V. Blood pressure variability and autonomic dysfunction. Curr Diab Rep. (2018) 18:1–14. doi: 10.1007/s11892-018-1108-z 30361834

[36] Asefa NG Neustaeter A Jansonius NM Snieder H. Autonomic dysfunction and blood pressure in glaucoma patients: the lifelines cohort study. Invest Ophthalmol Vis Sci. (2020) 61:25. doi: 10.1167/iovs.61.11.25, PMID: 32931573 PMC7500113

[37] Imai Y Aihara A Ohkubo T Nagai K Tsuji I Minami N. Factors that affect blood pressure variability. A community-based study in Ohasama, Japan. Am J Hypertens. (1997) 10:1281–9. doi: 10.1016/s0895-7061(97)00277-x 9397248

[38] Kim KI Nikzad N Quer G Wineinger NE Vegreville M Normand A. Real world home blood pressure variability in over 56,000 individuals with nearly 17 million measurements. Am J Hypertens. (2018) 31:566–73. doi: 10.1093/ajh/hpx221, PMID: 29365036 PMC5905665

[39] Bencivenga L De Souto BP Rolland Y Hanon O Vidal JS Cestac P. Blood pressure variability: a potential marker of aging. Ageing Res Rev. (2022) 80:101677. doi: 10.1016/j.arr.2022.101677 35738476

[40] Yoo JE Shin DW Han K Kim D Lee SP Jeong SM. Blood pressure variability and the risk of dementia: a nationwide cohort study. Hypertension. (2020) 75:982–90. doi: 10.1161/HYPERTENSIONAHA.119.14033 32148122

[41] Yoo JE Yoon JW Park HE Han K Shin DW. Blood pressure variability and the risk of fracture: a nationwide cohort study. J Clin Endocrinol Metab. (2022) 107:e1488–500. doi: 10.1210/clinem/dgab856, PMID: 34850029

