# Supplementary material for: Visit-to-visit variability in blood pressure and the risk of open-angle glaucoma in individuals without systemic hypertension: a nationwide population-based cohort study
Source: Front Med (Lausanne). 2024 Jan 10;10:1300778. doi: 10.3389/fmed.2023.1300778 (PMC10805885; doi:10.3389/fmed.2023.1300778)
Supplement: Supplementary file 1 [file Data_Sheet_1.PDF]

Supplementary Online Materials

Table S1. Baseline characteristics of the study participants, based on the SD quartiles for systolic blood pressure variability
Table S2. Baseline characteristics of the study participants, based on the CV quartiles for systolic blood pressure variability
Table S3. Baseline characteristics of the study participants, based on the SD quartiles for diastolic blood pressure variability
Table S4. Baseline characteristics of the study participants, based on the CV quartiles for diastolic blood pressure variability
Table S5. Subgroup analyses of the impact of systolic BP variability (SD) on the development of OAG.
Table S6. Subgroup analyses of the impact of systolic BP variability (VIM) on the development of OAG.
Table S7. The Korean Standard Classification of Disease codes for conditions related to secondary glaucoma

Table S1. Baseline characteristics of the study participants, based on the SD quartiles for systolic blood pressure variability

	Q1	Q2	Q3	Q4	*P*-value[a]
**N**	35220	35284	35177	35229	
**Age (y)**	54.7±8.2	53.5±7.4	55.0±8.3	57.8±9.3	<.0001
**Sex (male)**	19732 (56.0)	22835 (64.7)	21384 (60.8)	19948 (56.6)	<.0001
**BMI (kg/m^2)**	23.7±2.8	23.8±2.7	23.8±2.8	24.1±3.0	<.0001
**Mean SBP**	122.0±11.8	123.3±11.3	124.8±12.1	131.0±13.8	<.0001
**Mean DBP**	76.8±7.8	77.7±7.5	78.5±7.7	81.3±8.6	<.0001
**SBP variability**					
**CV (%)**	3.7±1.4	6.6±1.0	9.0±1.1	13.6±3.3	<.0001
**SD**	4.5±1.6	8.1±0.9	11.1±1.0	17.8±4.7	<.0001
**VIM**	4.9±2.0	8.6±1.7	11.6±2.1	16.9±4.4	<.0001
**DBP variability**					
**CV (%)**	7.1±3.9	8.3±3.7	9.5±4.1	12.3±5.3	<.0001
**SD**	5.4±3.0	6.4±2.8	7.4±3.2	10.0±4.4	<.0001
**VIM**	5.6±3.1	6.5±2.9	7.5±3.3	9.7±4.2	<.0001
**FPG**	96.4±22.4	96.6±21.8	97.5±23.2	99.7±26.3	<.0001
**Total cholesterol**	198.5±36.2	198.1±35.6	198.8±36.3	199.8±37.3	<.0001
**AST**	25.6±13.6	25.8±15.1	26.3±16.4	27.1±17.3	<.0001
**ALT**	24.5±17.9	25.2±19.7	25.3±20.8	25.6±20.0	<.0001
**GGT**	35.5±43.4	37.8±46.5	38.8±51.3	41.8±59.0	<.0001
**DM**	4341 (12.3)	4948 (14.0)	5378 (15.3)	6606 (18.8)	<.0001
**Hyperlipidemia**	10822 (30.7)	11073 (31.4)	11460 (32.6)	12914 (36.7)	<.0001
**Cataract**	2564 (7.3)	2042 (5.8)	2493 (7.1)	3506 (10.0)	<.0001
**CCI**					<.0001
**0**	13796 (39.2)	14860 (42.1)	13846 (39.4)	11745 (33.3)	
**1**	10043 (28.5)	10093 (28.6)	9830 (27.9)	9623 (27.3)	
**2**	5639 (16.0)	5425 (15.4)	5749 (16.3)	6322 (17.9)	
**≥3**	5742 (16.3)	4906 (13.9)	5752 (16.4)	7539 (21.4)	
**Current smoker**	6761 (19.2)	8218 (23.3)	7692 (21.9)	7025 (19.9)	<.0001
**Alcohol consumption**	3067 (8.7)	3354 (9.5)	3512 (10.0)	3861 (11.0)	<.0001
**Regular exercise**	3594 (10.2)	3230 (9.2)	3416 (9.7)	3682 (10.5)	<.0001
**Income (<10%)**	2432 (6.9)	2531 (7.2)	2722 (7.7)	3409 (9.7)	<.0001

Data are expressed as the mean ± SD, or as n (%).
[a]The *P*-value is derived from the analysis of variance and the Chi-square test.
SD, standard deviation; Q1–4, quartile 1–4; N, number; BMI, body mass index; SBP, systolic blood pressure; DBP, diastolic blood pressure; VIM, variability independent of the mean; CV, coefficient of variation; FPG, fasting plasma glucose; AST, aspartate aminotransferase; ALT, alanine aminotransferase; GGT, gamma glutamyl transferase; DM, diabetes mellitus; and CCI, Charlson comorbidity index

Table S2. Baseline characteristics of the study participants, based on the CV quartiles for systolic blood pressure variability

	**Q1**	**Q2**	**Q3**	**Q4**	***P*-value[a]**
**N**	35228	35179	35272	35231	
**Age (years)**	55.1±8.4	54.0±7.8	54.7±8.2	57.3±9.2	<.0001
**Sex (male)**	20445 (58.0)	22673 (64.5)	21638 (61.3)	19143 (54.3)	<.0001
**BMI (kg/m^2)**	23.9±2.7	23.9±2.8	23.8±2.8	23.9±3.0	.0008
**Mean SBP**	124.2±11.8	124.7±12.4	124.6±12.4	127.5±14.2	<.0001
**Mean DBP**	78.1±7.7	78.5±8.0	78.4±7.9	79.5±8.7	<.0001
**SBP variability**					
**CV (%)**	3.7±1.4	6.5±0.7	9.0±0.8	13.8±3.1	<.0001
**SD**	4.5±1.7	8.1±1.2	11.2±1.5	17.6±4.8	<.0001
**VIM**	4.7±1.8	8.4±1.1	11.5±1.3	17.3±4.0	<.0001
**DBP variability**					
**CV (%)**	7.1±3.9	8.2±3.7	9.5±4.0	12.4±5.2	<.0001
**SD**	5.5±3.0	6.4±3.0	7.4±3.2	9.9±4.4	<.0001
**VIM**	5.6±3.1	6.5±3.0	7.5±3.2	9.8±4.1	<.0001
**FPG**	97.0±22.9	97.2±22.5	97.6±24.0	98.4±24.7	<.0001
**Total cholesterol**	198.9±36.3	198.6±35.8	198.7±36.2	199.1±37.1	0.162
**AST**	25.8±13.8	26.1±15.7	26.2±16.2	26.7±16.8	<.0001
**ALT**	24.9±17.9	25.4±19.5	25.3±20.9	25.1±20.0	0.002
**GGT**	36.8±45.4	38.7±48.8	39.0±51.0	39.5±56.0	<.0001
**DM**	4635 (13.2)	5197 (14.8)	5417 (15.4)	6024 (17.1)	<.0001
**Hyperlipidemia**	11157 (31.7)	11266 (32.0)	11472 (32.5)	12374 (35.1)	<.0001
**Cataract**	2663 (7.6)	2196 (6.2)	2408 (6.8)	3338 (9.5)	<.0001
**CCI**					<.0001
**0**	13765 (39.1)	14459 (41.1)	14001 (39.7)	12022 (34.1)	
**1**	9957 (28.3)	9977 (28.4)	9888 (28.0)	9767 (27.7)	
**2**	5640 (16.0)	5497 (15.6)	5746 (16.3)	6252 (17.7)	
**≥3**	5866 (16.7)	5246 (14.9)	5637 (16.0)	7190 (20.4)	
**Current smoker**	6920 (19.6)	8068 (22.9)	7852 (22.3)	6856 (19.5)	<.0001
**Alcohol consumption**	3289 (9.3)	3439 (9.8)	3525 (10.0)	3541 (10.1)	0.006
**Regular exercise**	3630 (10.3)	3290 (9.4)	3399 (9.6)	3603 (10.2)	<.0001
**Income (<10%)**	2472 (7.0)	2491 (7.1)	2793 (7.9)	3338 (9.5)	<.0001

Data are expressed as the mean ± SD, or as n (%).

[a]The *P*-value is derived from the analysis of variance and the Chi-square test.

CV, coefficient of variation; Q1–4, quartile 1–4; N, number; BMI, body mass index; SBP, systolic blood pressure; DBP, diastolic blood pressure; VIM, variability independent of the mean; SD, standard deviation; FPG, fasting plasma glucose; AST, aspartate aminotransferase; ALT, alanine aminotransferase; GGT, gamma glutamyl transferase; DM, diabetes mellitus; and CCI, Charlson comorbidity index

Table S3. Baseline characteristics of the study participants, based on the SD quartiles for diastolic blood pressure variability

	Q1	Q2	Q3	Q4	*P*-value[a]
**N**	36043	34511	35130	35226	
**Age (y)**	54.8±8.2	55.2±8.5	54.1±7.7	56.9±9.1	<.0001
**Sex (male)**	20793 (57.7)	20128 (58.3)	22601 (64.3)	20377 (57.8)	<.0001
**BMI (kg/m^2)**	23.8±2.8	23.8±2.8	23.8±2.8	24.1±3.0	<.0001
**Mean SBP**	123.5±12.0	123.5±12.1	125.0±12.1	129.1±13.9	<.0001
**Mean DBP**	77.5±7.7	77.5±7.6	78.5±7.7	80.9±8.8	<.0001
**SBP variability**					
**CV (%)**	6.3±3.1	7.3±3.4	8.3±3.3	11.1±4.7	<.0001
**SD**	7.8±4.0	9.0±4.4	10.4±4.4	14.4±6.5	<.0001
**VIM**	8.1±4.1	9.4±4.4	10.6±4.4	13.9±5.9	<.0001
**DBP variability**					
**CV (%)**	4.2±2.1	7.5±0.9	10.3±1.4	15.4±3.6	<.0001
**SD**	3.2±1.6	5.7±0.4	8.0±0.8	12.4±3.0	<.0001
**VIM**	3.3±1.7	5.9±0.8	8.2±1.2	12.2±2.9	<.0001
**FPG**	97.0±21.9	97.1±23.5	97.4±23.3	98.8±25.3	<.0001
**Total cholesterol**	198.8±35.9	198.8±36.2	198.0±36.0	199.6±37.2	<.0001
**AST**	25.9±15.6	25.9±14.2	26.2±16.1	26.8±16.6	<.0001
**ALT**	25.0±20.3	24.8±18.4	25.5±20.1	25.4±19.6	<.0001
**GGT**	36.6±46.2	36.9±47.9	39.2±49.1	41.3±57.6	<.0001
**DM**	4857 (13.5)	4753 (13.8)	5503 (15.7)	6160 (17.5)	<.0001
**Hyperlipidemia**	11217 (31.1)	10937 (31.7)	11394 (32.4)	12721 (36.1)	<.0001
**Cataract**	2642 (7.3)	2556 (7.4)	2155 (6.1)	3252 (9.2)	<.0001
**CCI**					<.0001
**0**	14219 (39.5)	13407 (38.8)	14357 (40.9)	12264 (34.8)	
**1**	10201 (28.3)	9700 (28.1)	9976 (28.4)	9712 (27.6)	
**2**	5858 (16.3)	5593 (16.2)	5524 (15.7)	6160 (17.5)	
**≥3**	5765 (16.0)	5811 (16.8)	5273 (15.0)	7090 (20.1)	
**Current smoker**	7194 (20.0)	7154 (20.7)	8172 (23.3)	7176 (20.4)	<.0001
**Alcohol consumption**	3296 (9.1)	3320 (9.6)	3444 (9.8)	3734 (10.6)	<.0001
**Regular exercise**	3616 (10.0)	3456 (10.0)	3224 (9.2)	3626 (10.3)	<.0001
**Income (<10%)**	2656 (7.4)	2503 (7.3)	2730 (7.8)	3205 (9.1)	<.0001

Data are expressed as the mean ± SD, or as n (%).

[a]The *P*-value is derived from the analysis of variance and the Chi-square test.

SD, standard deviation; Q1–4, quartile 1–4; N, number; BMI, body mass index; SBP, systolic blood pressure; DBP, diastolic blood pressure; VIM, variability independent of the mean; CV, coefficient of variation; FPG, fasting plasma glucose; AST, aspartate aminotransferase; ALT, alanine aminotransferase; GGT, gamma glutamyl transferase; DM, diabetes mellitus; and CCI, Charlson comorbidity index

Table S4. Baseline characteristics of the study participants, based on the CV quartiles for diastolic blood pressure variability

	Q1	Q2	Q3	Q4	*P*-value[a]
**N**	35228	35179	35241	35262	
**Age (years)**	55.2±8.3	54.9±8.4	54.4±7.9	56.6±9.1	<.0001
**Sex (male)**	20958 (59.5)	21311 (60.6)	22172 (62.9)	19458 (55.2)	<.0001
**BMI (kg/m²)**	24.0±2.8	23.8±2.8	23.8±2.8	23.9±2.9	<.0001
**Mean SBP**	126.2±12.4	124.1±11.7	124.9±13.0	125.9±13.8	<.0001
**Mean DBP**	79.4±7.9	78.0±7.2	78.4±8.3	78.5±8.7	<.0001
**SBP variability**					
**CV (%)**	6.3±3.2	7.2±3.3	8.3±3.5	11.1±4.7	<.0001
**SD**	8.0±4.2	9.0±4.3	10.5±4.7	14.0±6.5	<.0001
**VIM**	8.0±4.0	9.3±4.2	10.7±4.4	14.0±5.9	<.0001
**DBP variability**					
**CV (%)**	4.0±2.0	7.4±0.6	10.2±1.0	15.6±3.4	<.0001
**SD**	3.2±1.6	5.8±0.7	8.0±1.2	12.2±3.2	<.0001
**VIM**	3.2±1.6	5.9±0.5	8.1±0.8	12.3±2.7	<.0001
**FPG**	97.7±22.6	97.4±24.0	97.5±23.9	97.7±23.7	0.258
**Total cholesterol**	199.7±36.2	198.6±36.1	198.2±36.1	198.8±36.9	<.0001
**AST**	26.2±15.7	26.0±15.2	26.2±15.6	26.4±16.2	0.010
**ALT**	25.5±20.4	25.0±18.5	25.4±20.0	24.8±19.6	<.0001
**GGT**	38.5±49.2	37.6±48.3	39.1±49.7	38.7±54.3	0.001
**DM**	5056 (14.4)	5070 (14.4)	5483 (15.6)	5664 (16.1)	<.0001
**Hyperlipidemia**	11454 (32.5)	11186 (31.8)	11497 (32.6)	12132 (34.4)	<.0001
**Cataract**	2673 (7.6)	2481 (7.1)	2299 (6.5)	3152 (8.9)	<.0001
**CCI**					<.0001
**0**	13756 (39.0)	13862 (39.4)	14121 (40.1)	12508 (35.5)	
**1**	9833 (27.9)	9920 (28.2)	10 032 (28.5)	9804 (27.8)	
**2**	5788 (16.4)	5592 (15.9)	5627 (16.0)	6128 (17.4)	
**≥3**	5851 (16.6)	5805 (16.5)	5461 (15.5)	6822 (19.3)	
**Current smoker**	7171 (20.4)	7526 (21.4)	8053 (22.9)	6946 (19.7)	<.0001
**Alcohol consumption**	3510 (10.0)	3459 (9.8)	3408 (9.7)	3417 (9.7)	0.524
**Regular exercise**	3612 (10.3)	3463 (9.8)	3357 (9.5)	3490 (9.9)	0.015
**Income (<10%)**	2586 (7.3)	2557 (7.3)	2803 (8.0)	3148 (8.9)	<.0001

Data are expressed as the mean ± SD, or as n (%).

[a]The *P*-value is derived from the analysis of variance and the Chi-square test.

CV, coefficient of variation; Q1–4, quartile 1–4; N, number; BMI, body mass index; SBP, systolic blood pressure; DBP, diastolic blood pressure; VIM, variability independent of the mean; CV, coefficient of variation; SD, standard deviation; FPG, fasting plasma glucose; AST, aspartate aminotransferase; ALT, alanine aminotransferase; GGT, gamma glutamyl transferase; DM, diabetes mellitus; and CCI, Charlson comorbidity index

Table S5. Subgroup analyses of the impact of systolic BP variability (SD) on the development of OAG.

	Q1–Q3	Q4	HR (95% CI)	*P*-value	*P* for interaction
**Age**					0.007
**<60 years**	568/80573	186/21449	1.18 (0.99-1.39)	0.061	
**≥60 years**	448/25108	219/13780	0.85 (0.72-1.01)	0.061	
**Sex**					0.694
**Female**	395/41730	179/15281	1.02 (0.85-1.22)	0.831	
**Male**	621/63951	226/19948	0.97 (0.83-1.14)	0.733	
**BMI (kg/m^2)**					0.158
**<25**	695/72622	246/22477	0.93 (0.80-1.08)	0.357	
**≥25**	321/33059	159/12752	1.11 (0.91-1.34)	0.301	
**DM**					0.967
**No**	850/91014	319/28623	0.99 (0.87-1.13)	0.899	
**Yes**	166/14667	86/6606	1.00 (0.77-1.30)	0.985	
**Hyperlipidemia**					0.221
**No**	665/72326	232/22315	0.94 (0.80-1.09)	0.401	
**Yes**	351/33355	173/12914	1.08 (0.90-1.31)	0.389	
**Cataract**					0.167
**No**	859/98582	334/31723	1.03 (0.90-1.18)	0.645	
**Yes**	157/7099	71/3506	0.83 (0.63-1.10)	0.196	
**Current smoker**					0.218
**No**	827/83010	347/28204	1.03 (0.90-1.17)	0.709	
**Yes**	189/22671	58/7025	0.84 (0.62-1.13)	0.244	
**Alcohol consumption**					0.604
**No**	910/95748	361/31368	1.00 (0.88-1.14)	0.958	
**Yes**	106/9933	44/3861	0.91 (0.64-1.29)	0.598	
**Regular exercise**					0.376
**No**	886/95441	344/31547	0.97 (0.85-1.11)	0.669	
**Yes**	130/10240	61/3682	1.13 (0.83-1.53)	0.442	
**Income (<10%)**					0.904
**No**	932/97996	361/31820	0.99 (0.87-1.12)	0.880	
**Yes**	84/7685	44/3409	1.01 (0.70-1.46)	0.941	

Note: Hazard ratios were calculated based on the multivariate Cox regression after adjustment for variables belonging to model 2.

BP, blood pressure; SD, standard deviation; Q1–4, quartile 1–4; HR, hazard ratio; CI, confidence interval; BMI, body mass index; DM, diabetes mellitus

Table S6. Subgroup analyses of the impact of systolic BP variability (VIM) on the development of OAG.

	**Q1-Q3**	**Q4**	**HR (95% CI)**	***P*-value**	***P* for interaction**
**Age**					0.082
**<60 y**	559/78910	195/23112	1.18 (1.00-1.39)	0.048	
**≥60 y**	463/26773	204/12115	0.96 (0.81-1.13)	0.627	
**Sex**					0.216
**Female**	400/40334	174/16677	0.98 (0.82-1.17)	0.802	
**Male**	622/65349	225/18550	1.13 (0.97-1.32)	0.110	
**BMI (kg/m^2)**					0.287
**<25**	681/71018	260/24081	1.02 (0.88-1.17)	0.827	
**≥25**	341/34665	139/11146	1.16 (0.95-1.41)	0.142	
**DM**					0.614
**No**	847/89955	322/29682	1.05 (0.92-1.19)	0.476	
**Yes**	175/15728	77/5545	1.13 (0.86-1.48)	0.368	
**Hyperlipidemia**					0.899
**No**	648/71367	249/23274	1.07 (0.92-1.24)	0.373	
**Yes**	374/34316	150/11953	1.05 (0.87-1.27)	0.596	
**Cataract**					0.834
**No**	867/98294	326/32011	1.07 (0.94-1.22)	0.308	
**Yes**	155/7389	73/3216	1.03 (0.78-1.37)	0.810	
**Current smoker**					0.398
**No**	834/82757	340/28457	1.09 (0.96-1.23)	0.199	
**Yes**	188/22926	59/6770	0.95 (0.71-1.27)	0.717	
**Alcohol consumption**					0.477
**No**	916/95169	355/31947	1.05 (0.93-1.19)	0.458	
**Yes**	106/10514	44/3280	1.20 (0.84-1.71)	0.311	
**Regular exercise**					0.569
**No**	881/95292	349/31696	1.08 (0.95-1.22)	0.244	
**Yes**	141/10391	50/3531	0.97 (0.71-1.35)	0.874	
**Income (<10%)**					0.943
**No**	935/97822	358/31994	1.06 (0.94-1.20)	0.341	
**Yes**	87/7861	41/3233	1.08 (0.74-1.56)	0.697	

Note: Hazard ratios were calculated based on the multivariate Cox regression after adjustment for variables belonging to model 2

BP, blood pressure; VIM, variability independent of the mean; Q1--4, quartile 1--4; HR, hazard ratio; CI, confidence interval; BMI, body mass index; DM, diabetes mellitus

Table S7. The Korean Standard Classification of Disease codes for conditions related to secondary glaucoma

Type of Condition	Eligible KCD Diagnosis Code
Endophthalmitis	H45.1, H44.0, H44.1
Retinal artery occlusion	H34.0, H34.1, H34.2
Retinal vein occlusion	H34.8, H34.9
Hemorrhage of retina and vitreous	H35.6, H43.1, H45.0
Retinal detachment/ Retinal break	H35.0,36.0, E10.3, E11.3, E12.3, E13.3, E14.3
Chorioretinal inflammation	H30.0, H30.1, H30.2, H30.8, H 30.9, H 32.0
Choroidal hemorrhage/ rupture	H31.3, H31.4
Degenerative myopia	H44.2

KCD, Korean Standard Classification of Disease
